# Effects of Amorphous Poly Alpha Olefin (APAO) and Polyphosphoric Acid (PPA) on the Rheological Properties, Compatibility and Stability of Asphalt Binder

**DOI:** 10.3390/ma14092458

**Published:** 2021-05-10

**Authors:** Xiaoguang Pei, Weiyu Fan

**Affiliations:** State Key Laboratory of Heavy Oil Processing, China University of Petroleum, Qingdao 266580, China; b15030089@s.upc.edu.cn

**Keywords:** composite modified asphalt binder, rheological properties, storage stability, microstructure, amorphous poly alpha olefin, polyphosphoric acid

## Abstract

High production costs and poor storage stability have become important constraints in the manufacture of modified asphalt binder. To simplify the production process and reduce the production cost, amorphous poly alpha olefin (APAO) and polyphosphoric acid (PPA) were applied to prepare highly stable modified asphalt binder. The influence of APAO/PPA on the temperature sensitivity, rheological property, storage stability, compatibility and microstructure of neat binder were studied by rotational viscosity (RV), dynamic shear rheometer (DSR), bending beam rheometer (BBR) and Fourier transform infrared (FTIR) spectroscopy. The results show that the incorporation of APAO/PPA reduced the temperature sensitivity of neat binder. The combined effect of APAO/PPA contributed to the improvement in deformation resistance, which was evidenced by the increase in failure temperature and percent recovery. However, the compound modification of APAO/PPA decreased the binder’s low-temperature performance. APAO strengthened the fatigue resistance of the binder, while PPA reduced the anti-fatigue performance. Composite modified asphalt binder with superior storage stability could be prepared, which was confirmed by the desired Cole–Cole plots and fluorescence imaging. Furthermore, chemical and physical reactions occurred during the APAO/PPA modification process. Overall, 2 wt.% (weight percentage) APAO and 1.5 wt.% PPA are recommended for the production of modified asphalt binder with remarkable rheological performance and storage stability.

## 1. Introduction

Asphalt pavement has become the main expressway pavement structure in China due to its characteristics of superior mechanical strength, low noise, driving safety and ease of mechanized construction. However, a variety of pavement stresses such as ruts, cracks, loosening and water damage have emerged due to the rapid increase in heavy traffic and the fluctuations caused by extreme climate. To resolve these issues, researchers have adopted various methods to improve the performance and longevity of asphalt pavement. Polymer modification is the most commonly used method to provide asphalt binder with a wide operating temperature range.

Depending on the polymer modifier types, thermoplastic elastomers and plastomers are the two main categories [1]. Thermoplastic elastomers, specifically styrene–butadiene–styrene (SBS), represent the most successful asphalt modifiers to date. Such modifiers can effectively enhance the resistance of asphalt binder against rutting, fatigue cracking and low-temperature cracking. However, the main drawback of these polymers is their partial miscibility with asphalt binder, which leads to poor storage stability of polymer modified binder. Plastomers, such as polyethylene (PE) and ethylene–vinyl–acetate (EVA), can significantly improve the rutting resistance of binder, but the improvement of low-temperature anti-cracking properties is limited. Its incompatibility with asphalt binder also results in severe separation between polymer and asphalt binder, which is undesirable and restricts its application [2]. Moreover, these polymers must be blended with asphalt binder by a colloid mill or high-speed shearing equipment [3,4,5]. Equipment investment and high shearing temperatures increase the production cost of modified asphalt binder [6]. In addition, these modified asphalt binders can only be stored for a short period of time and therefore must be used as soon as possible to avoid undesirable phase separation. Although the application of a stabilizer can improve the storage stability of the modified binder, it also leads to an increase in production cost. Therefore, high production cost and poor storage stability are significant constraints currently faced in the manufacture of modified asphalt binder. To reduce the production cost without compromising on the properties of modified asphalt binder, the development of new types of modifiers to replace colloid mills and stabilizers and allow for lower processing temperatures and simplified production processes is of significant research interest in the field.

Amorphous poly alpha olefin (APAO) is a low molecular weight amorphous plastic material, which is highly compatible with asphalt binder [7,8]. It is formed by the copolymerization of α-olefin and has the characteristics of high fluidity, low crystallinity and high randomness. Previous studies have shown that APAO can be completely dissolved in asphalt binder when the APAO content is no more than 6 wt.% [7,9]. It has also been found that APAO modified binder with high stability can be processed without shearing and stabilizer. Kong et al. [10] showed that the processing temperature of APAO modified binder can be controlled at 165 °C, which is lower than the processing temperature of 180 °C for SBS modified binder. These results demonstrate that APAO is capable of producing modified asphalt binder at a lower processing temperature without shearing and stabilizer. Regarding the impact on asphalt binder performance, numerous studies on the mechanical properties of APAO modified binders have been conducted. Wei et al. [7,9] showed that APAO enhanced the elastic property and lowered the temperature susceptibility of binder, but had adverse effects on the creep stiffness and creep rate. Yan et al. [11] and Liu et al. [12] determined that APAO improved the high-temperature performances, storage stability and aging resistance of waste tire rubber (WTR) modified binder. However, its effect on low-temperature properties remains uncertain and is highly debated. Liu et al. [13] concluded that SBS/APAO modified binder had superior high- and intermediate-temperature properties and storage stability in comparison to SBS modified binder, while its low-temperature performance was equivalent to that of SBS modified binder. You et al. [14] found that APAO enhanced the high- and low-temperature properties as well as storage stability of terminal blend rubberized binder (TB). Yan et al. [15] showed that the incorporation of APAO strengthened the elastic recovery and aging resistance as well as low-temperature cracking resistance of EVA modified binder. Moreover, the results demonstrated that APAO was a good stabilizer to improve the stability of asphalt binder. Evidently, APAO is a modifier that satisfies the requirement due to its superior compatibility with binder, simple modification process and excellent modification effect. In addition, APAO has a comparable price to SBS. In order to further reduce the cost, it is important to choose another modifier that has good compatibility with asphalt binder and combine it with APAO for the modification of neat binder.

Polyphosphoric acid (PPA), a mineral acid, has good compatibility with asphalt binder and has been widely applied in asphalt binder modification [16,17]. The preparation process of PPA modified asphalt binder is relatively simple, which involves uniform mixing through stirring at 150–160 °C instead of high-speed shearing [18,19]. PPA does not separate from the asphalt phase when it modifies the asphalt binder in the absence of a stabilizer [20]. Previous research has shown that a small amount of PPA can markedly improve the Superpave performance grade (PG) of asphalt binder [18,21]. The useful temperature interval (UTI) is extended by PPA due to its characteristics of increasing the high-temperature stiffness without decreasing the low-temperature flexibility [22,23,24,25]. PPA was also adopted to modify asphalt binder with another polymer to strengthen its rheological behavior and reduce the cost [26,27,28,29]. Moreover, PPA can enhance the compatibility between polymer and asphalt binder during the composite modification process [27,30]. Overall, PPA is a desirable modifier that has similar characteristics to APAO in terms of good compatibility and a simple modification process.

The literature review shows that both APAO and PPA can produce highly stable modified asphalt binder at relatively low processing temperatures without shearing and stabilizer. The recent studies on APAO and PPA modification are summarized in Table 1. Thus, the combination of APAO and PPA can provide some enlightenment for improving the performance of modified asphalt binder and simplifying the process technology. To date, investigations regarding APAO/PPA modified binders have not been published, and the modification effect and mechanism of this unique modification are still unknown. Hence, systematic and comprehensive studies on the interaction between APAO/PPA and asphalt binder are still needed.

This study aims to explore the compound modification effect of APAO and PPA on the base binder. The conventional properties, rheological behavior, storage stability, compatibility and microstructure of composite modified binders were evaluated. Furthermore, the modification effects of APAO/PPA compound modification were compared with those of APAO modification alone and PPA modification alone. The optimal proportions of APAO and PPA were also determined to provide basic parameters for industrial production. Finally, variations in functional groups of asphalt binder were revealed by FTIR spectroscopy measurements.

## 2. Experiment and Methods

### 2.1. Materials

Neat binder produced from Suizhong crude oil was adopted for modification, and its penetration grade was 80/100 according to the ASTM standard. Its basic specifications are presented in Table 2. PPA was supplied by Macklin in China. The phosphorous pentoxide (P_2_O_5_) concentration was greater than 85 wt.% and the density was 2.05 g/cm^3^. The APAO-828 compound applied in this study was manufactured by Evonik Degussa of Germany. The key parameters of APAO-828 are summarized in Table 3.

### 2.2. Specimen Preparation

**Figure 1:**
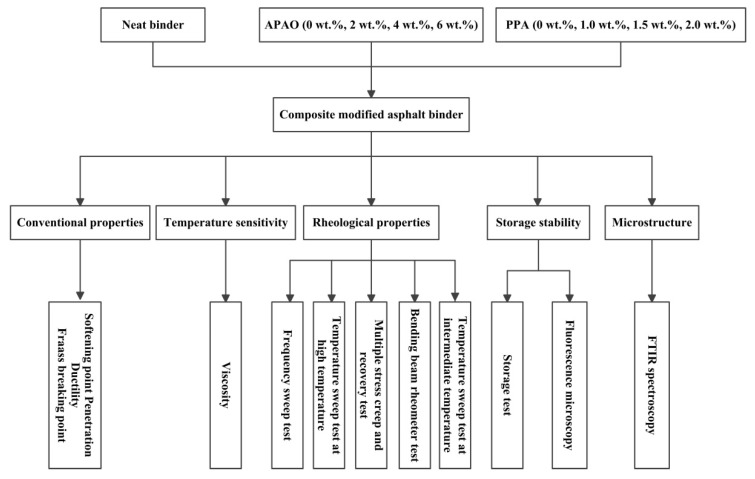
Flowchart of the experimental program.

The compound modified binders were prepared by a high-speed stirring approach without a stabilizer. First, the neat binder was preheated to 165 °C in an iron cylinder and then APAO was slowly added. The temperature of the iron cylinder was maintained at 165 °C and the blend was stirred at 2000 rpm for 30 min. Then, PPA was poured into this cylinder and stirred for another 30 min. Subsequently, the prepared samples were subjected to analysis. Different contents of APAO and PPA were mixed together to prepare different modified binders. Four percentages of APAO (0 wt.%, 2 wt.%, 4 wt.% and 6 wt.%) and PPA (0 wt.%, 1.0 wt.%, 1.5 wt.% and 2.0 wt.%) (by the weight of neat binder) were employed in this research. For simplicity, the modified binders with various concentrations of APAO and PPA are labeled. The composition and corresponding abbreviations of all modified binders are listed in Table 4. The detailed technical flowchart of this study is shown in Figure 1.

### 2.3. Test Methods

#### 2.3.1. Conventional Property Tests

The conventional property tests, including penetration at 25 °C (ASTM D 5), softening point (ASTM D 36), ductility at 10 °C (ASTM D113) and the Fraass breaking point (BS EN 12593) [38], were carried out.

The viscosity of the asphalt binder was tested with a Brookfield rotational viscometer according to ASTM D 4402. The measurements were conducted from 135 °C to 175 °C to assess its construction workability and temperature sensitivity.

#### 2.3.2. Rheological Performance Tests

(1) DSR Test

A dynamic shear rheometer (DSR) with parallel plate geometry was applied to study the rheological behavior of asphalt binder at high and intermediate temperatures according to ASTM D 7175 [39]. Plates of a 25 mm diameter spaced 1 mm apart were selected for samples to detect the shear storage modulus G′ and shear loss modulus G″ at high temperatures. The linear viscoelastic range of each sample was identified by a strain sweep test. Each test was repeated twice to confirm a good repeatability of the data.

The frequency sweep test was carried out from 0.1 rad/s to 100 rad/s at 60 °C to obtain dynamic shear moduli. The temperature sweep test was performed at a fixed frequency of 10 rad/s and the temperature was raised from 30 °C to 90 °C with an interval of 5 °C to evaluate the high-temperature performance. Samples aged in the rolling thin film oven test (RTFOT) and pressure aging vessel (PAV) were subject to a temperature sweep test from 10 °C to 40 °C to investigate the fatigue performance.

(2) MSCR test

The multiple stress creep and recovery (MSCR) test, according to ASTM D 7405 [40], was performed to obtain the non-recoverable creep compliance and percent recovery, which could accurately reflect the mechanical response of the polymer modified asphalt binder. The test was conducted at 60 °C by applying shear stresses of 0.1 kPa and 3.2 kPa, respectively. The RTFOT aged samples were applied in this experiment.

(3) BBR test

The bending beam rheometer (BBR) test was employed to measure the low-temperature rheological performance of asphalt binder, according to ASTM D6648 [41]. Two main factors, creep stiffness (S) and creep rate (m-value), which indicate the low-temperature crack resistance, were obtained under a 60 s loading. The stiffness is the ratio of stress to the total strain under a certain duration and temperature. The m-value reflects the relaxation rate of asphalt binders. RTFOT-PAV aged samples were characterized at −6 °C, −12 °C and −18 °C, respectively.

#### 2.3.3. Storage Test

The storage stability test was conducted to identify the phase separation degree between polymer and asphalt binder. According to ASTM D7173 [42], approximately 50 g of compound modified binder were poured into a cigar tube with a diameter and height of 2.5 cm and 14 cm, respectively. Then, the tube was sealed and placed vertically in an oven at 163 °C. After storage for 48 h, the tube was taken out of the oven and put into the refrigerator to cool down. The cooled tube was divided into three equal parts and the softening points of the upper and lower parts were tested.

#### 2.3.4. Microstructure

(1) ATR-FTIR test

The functional groups of the asphalt binder were determined by attenuated total reflection Fourier transform infrared (ATR-FTIR) spectroscopy (Cary 630 FTIR Microscope, Agilent, Santa Clara, CA, USA). The infrared spectrum was recorded in the wavenumber range of 650 cm^−1^ to 4000 cm^−1^.

(2) FM test

The dispersion of APAO and PPA in the binder was observed by a fluorescence microscope (FM) (Olympus, Tokyo, Japan). The magnification of the observed images was 400×.

## 3. Results and Discussion

### 3.1. Conventional Properties

**Figure 2:**
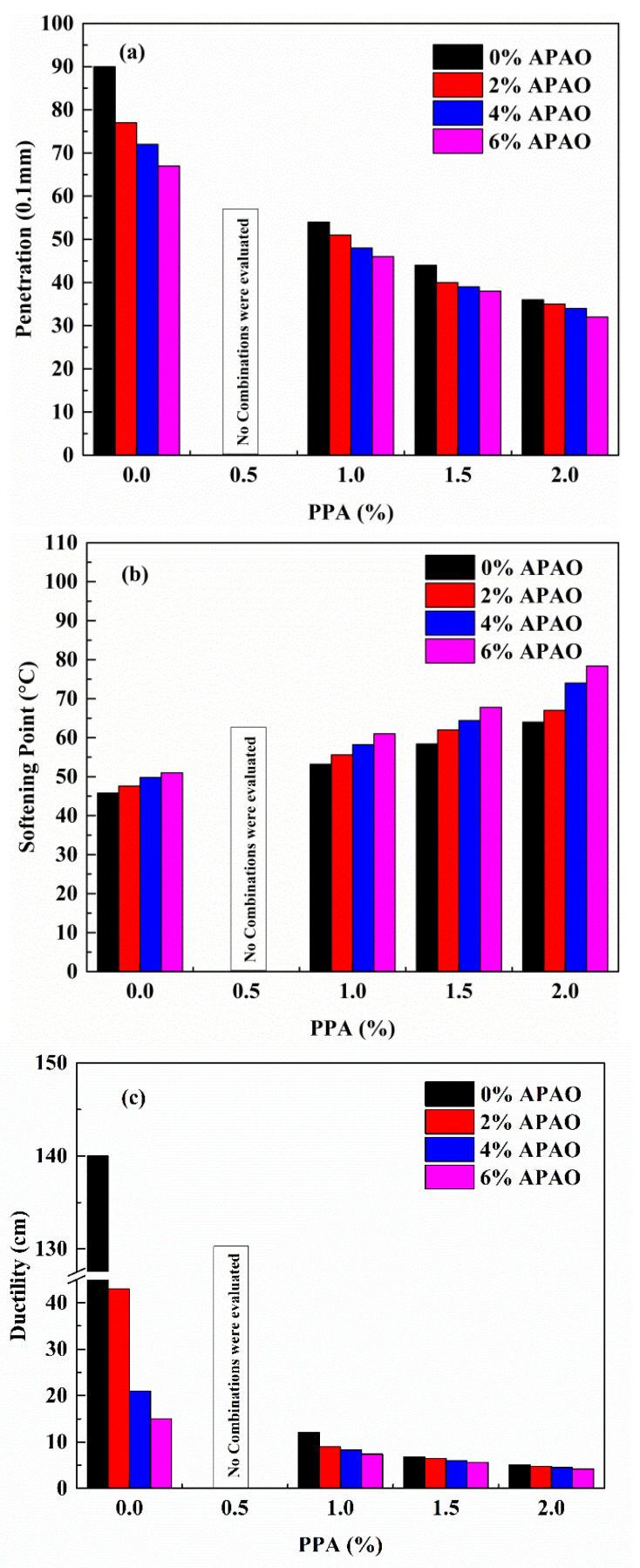

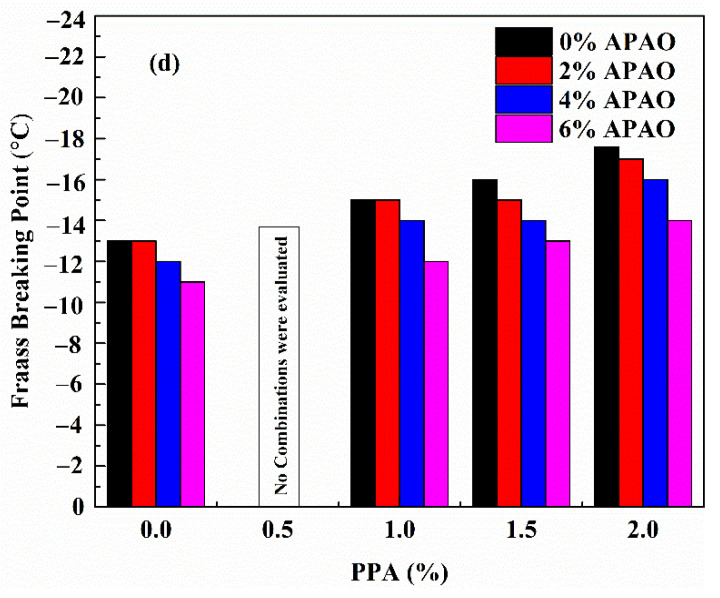
Conventional properties of all specimens: (**a**) penetration; (**b**) softening point; (**c**) ductility; (**d**) Fraass breaking point.

The effects of APAO/PPA on conventional characteristics, such as the penetration, softening point, ductility and Fraass breaking point of the modified binder are shown in Figure 2a–d. It is evident from Figure 2a that the penetration of asphalt binder declines with increases in APAO content when the PPA concentration is constant. When APAO content increases from 0 wt.% to 6 wt.%, the reduction in penetration is 25.6%, 14.8%, 13.6% and 11.1% when the PPA content is 0 wt.%, 1.0 wt.%, 1.5 wt.% and 2.0 wt.%, respectively. This indicates that when APAO concentration increases by 6 wt.%, the reduction in penetration decreases with the increase in PPA content. Moreover, the reduced level of penetration through the incorporation of APAO is lower than that from PPA. The compound modification of APAO and PPA leads to a minimum penetration of 32 (0.1 mm), which is a reduction of 58 (0.1 mm) compared with the base binder. Figure 2b displays an ascending trend of softening point with increases in APAO or PPA concentration, indicating that both APAO and PPA could reinforce the high-temperature performances of asphalt binder. When the PPA dosage is 0 wt.%, 1.0 wt.%, 1.5 wt.% and 2.0 wt.%, a 6 wt.% increase in APAO leads to an 11.4%, 14.7%, 16.1% and 22.5% increase in softening point, respectively. This shows that the increment in the softening point increases with increases in PPA content when the APAO increases by 6 wt.%. Similarly, when the APAO dosage is 0 wt.%, 2 wt.%, 4 wt.% and 6 wt.%, the softening point increases by 39.7%, 40.8%, 49.5% and 53.7%, when the PPA content increases by 2.0 wt.%. It is noteworthy that the increase in softening point caused by PPA is greater than that by APAO.

Figure 2c,d present the variations of ductility at 10 °C and Fraass breaking point. Figure 2c shows that APAO significantly decreases the ductility of asphalt binder. When APAO increases by 6 wt.%, the reduction in ductility is 89.3%, 38.8%, 17.6% and 17.4%, when the PPA content is 0 wt.%, 1.0 wt.%, 1.5 wt.% and 2.0 wt.%, respectively. This indicates that when the APAO concentration increases by 6 wt.%, the reduction in ductility decreases with an increase in PPA content. After the incorporation of PPA, the ductility is further reduced on the basis of APAO. The compound modification of APAO and PPA results in a marked reduction in ductility, indicating that the low-temperature performance decreases. The literature [43,44] reveals that the incorporation of APAO is detrimental to the low-temperature properties of a mixture. The Fraass breaking point denotes the turning point of asphalt binder, where it transforms from a viscoelastic state to a brittle state. Figure 2d shows that APAO is harmful to the Fraass breaking point, while PPA is beneficial to the Fraass breaking point. When the PPA dosage is 0 wt.%, 1.0 wt.% and 2.0 wt.%, a 6 wt.% increase in APAO leads to a 15.4%, 20.0% and 22.2% increase in Fraass breaking point, respectively. Meanwhile, a 2.0 wt.% increase in PPA content contributes to a decrease by 38.5%, 30.8% and 27.3% in the Fraass breaking point, when the APAO content is 0 wt.%, 2 wt.% and 6 wt.%. The results show that PPA has a greater effect on the Fraass breaking point. The combined effect of APAO and PPA leads to a decrease in the Fraass breaking point, indicating that APAO/PPA could reduce the turning point of the binder’s brittle state. In summary, the compound modification of APAO and PPA could enhance the high-temperature properties but decrease the low-temperature flexibility.

### 3.2. Temperature Sensitivity

**Figure 3:**
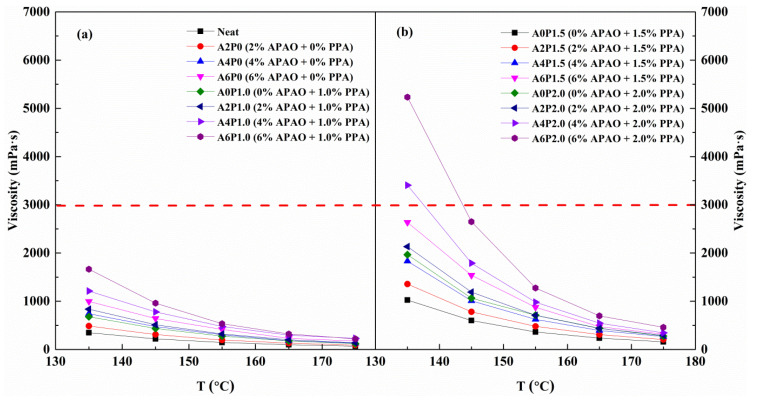
Variations in viscosity with temperature for all specimens: (**a**) composite modified binders with 0 wt.% and 1.0 wt.% PPA combined with 0-6 wt.% APAO; (**b**) composite modified binders with 1.5 wt.% and 2.0 wt.% PPA combined with 0-6 wt.% APAO.

The effects of APAO and PPA on changes in viscosity are shown in Figure 3. Notably, the viscosity increases as the content of APAO and PPA increases. The viscosity increases by 1.8 times when the APAO proportion increases from 0 wt.% to 6 wt.%, and the viscosity improves by 4.4 times as the PPA content increases by 2.0 wt.%. The results demonstrate that PPA has a greater effect on the increase in viscosity than APAO. Viscosity is a vital indicator to reveal the rheological behavior of asphalt binder, whereby SHRP requires that the 135 °C viscosity should be less than 3.0 Pa·s to guarantee construction workability. From Figure 3a,b, when the PPA proportion remains at 1.0 wt.% and 1.5 wt.%, and the APAO content reaches 6 wt.%, the viscosity meets the SHRP requirement. However, the viscosity exceeds 3.0 Pa·s when the PPA content increases to 2.0 wt.% and APAO content reaches 4 wt.%. Thus, to ensure the workability of asphalt binder, the APAO content could range from 0 wt.% to 6 wt.% when the PPA content is less than 2.0 wt.%, and the APAO proportion should not exceed 4 wt.% when the PPA content reaches 2.0 wt.%. As a result, two types of modified binders are chosen: one is 1.5 wt.% PPA combined with different contents of APAO, and the other is 2 wt.% APAO mixed with different concentrations of PPA.

**Figure 4:**
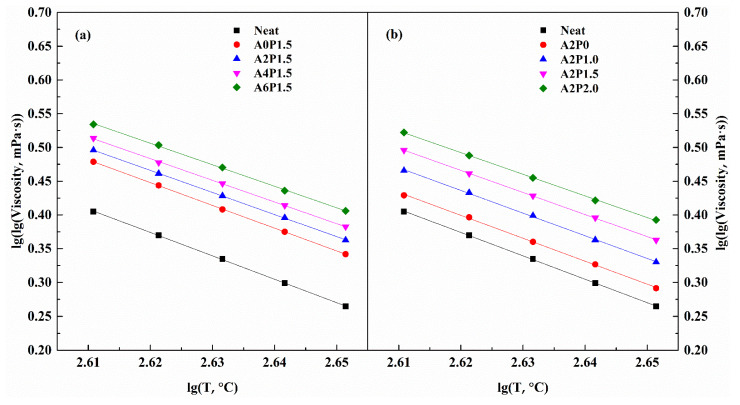
Viscosity−temperature relationship for composite modified binders: (**a**) composite modified binders with various contents of APAO; (**b**) composite modified binders with various contents of PPA.

The temperature sensitivity is an important rheological index for asphalt binder, which is usually determined by the viscosity–temperature curve. The asphalt binder’s temperature sensitivity determines the characteristics of an asphalt pavement. Low temperature sensitivity of asphalt binder indicates better asphalt pavement performance. Figure 4a,b display the variations in viscosity of composite modified binder at different temperatures. Curve fitting is performed to show the correlation between viscosity and temperature and, from which, the slopes and coefficient of determinations (R^2^) are obtained, as listed in Table 5. The R^2^ value is greater than 0.99, which indicates that lg(lg(viscosity)) has a good linear correlation with lg(T). A lower slope value indicates that the asphalt binder is less susceptible to temperature changes. In comparison to neat binder, the modified asphalt binders A0P1.5 and A2P0 have lower slope values. Notably, the slope value of A2P0 is higher than that of A0P1.5, which indicates that the enhancement against temperature sensitivity of PPA is greater than that of APAO. It can be seen that the slope value of the fitted curve becomes smaller with increases in APAO or PPA content, indicating that APAO and PPA contribute to the reduction in temperature sensitivity. Furthermore, the mixing and compaction temperatures of all samples are determined according to AASHTO T312 viscosity requirements of (0.17 ± 0.02) Pa·s and (0.28 ± 0.02) Pa·s [45]. Table 6 shows the mixing and compaction temperatures for all tested samples. It is clear that the mixing temperature and compaction temperature of composite modified binder increase with increases in APAO and PPA content. The difference between the mixing temperature and the compaction temperature of neat binder is 11.21 °C, and the temperature difference increases with increasing content of APAO and PPA. The temperature difference is 13.40 °C for A6P1.5 and 13.17 °C for A2P2.0. This indicates that a higher temperature is needed to reach the same viscosity, which demonstrates that APAO and PPA reduce the temperature sensitivity of asphalt binder. Therefore, the incorporation of APAO and PPA has the potential to strengthen the asphalt pavement performance.

### 3.3. Rheological Properties

#### 3.3.1. Frequency Sweep Tests

Asphalt binder is a viscoelastic material whose stress–strain characteristics depend on temperature and time. The mechanical and viscoelastic behavior of asphalt binder varies with loading frequency. Thus, the effects of compound modification of APAO and PPA on the rheological behavior of base binder were investigated by frequency sweep tests.

**Figure 5:**
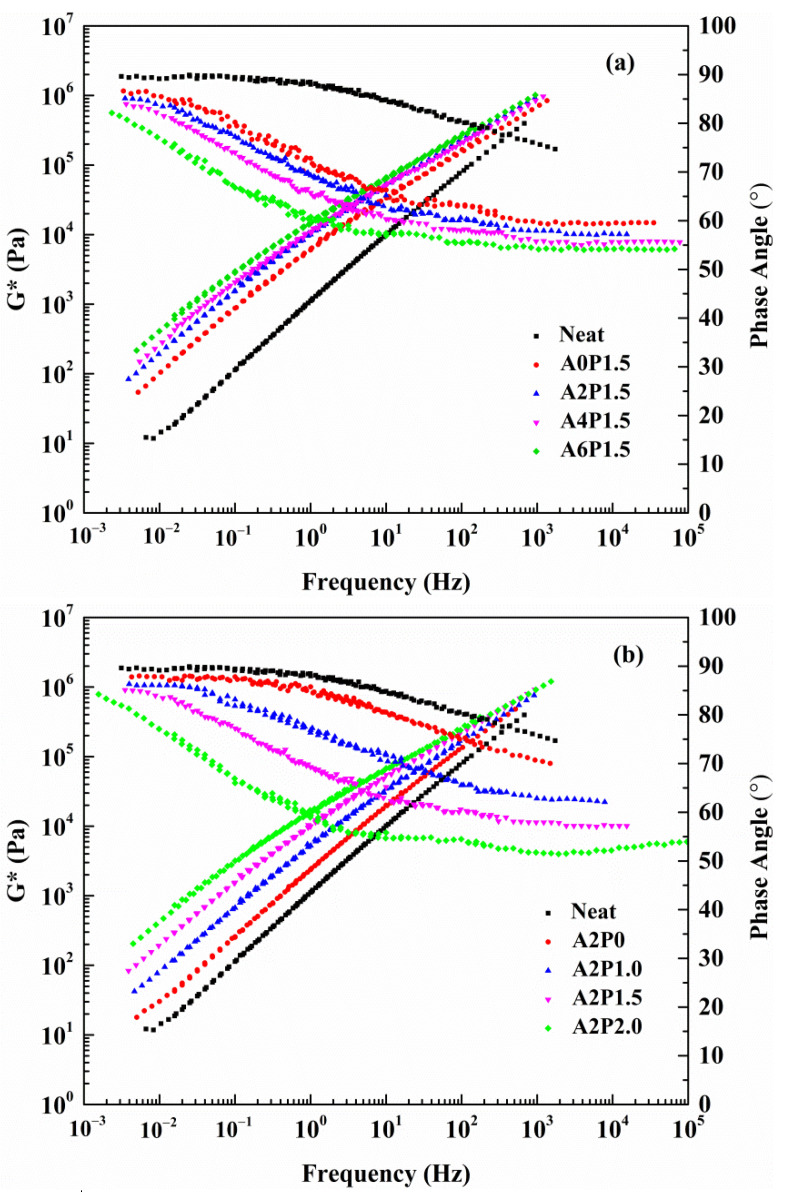
Complex modulus and phase angle master curves of composite modified binders: (**a**) composite modified binders with various contents of APAO; (**b**) composite modified binders with various contents of PPA.

Figure 5 shows the master curves of complex modulus (G*) and phase angle (δ) for the composite modified binders. Increases in the G* values and decreases in the δ values can be observed for all the tested binders. The complex modulus curve is elevated over the whole frequency range and the phase angle curve is lowered correspondingly. The increasing content of APAO and PPA causes an increase in G* values and a decrease in δ values, which indicates an increase in the elastic behavior of the asphalt binder. Moreover, G* values increase significantly at lower frequencies (higher temperatures) and increase slightly at higher frequencies (lower temperatures). This indicates that the incorporation of APAO and PPA improves the asphalt binder’s high-temperature performance.

**Figure 6:**
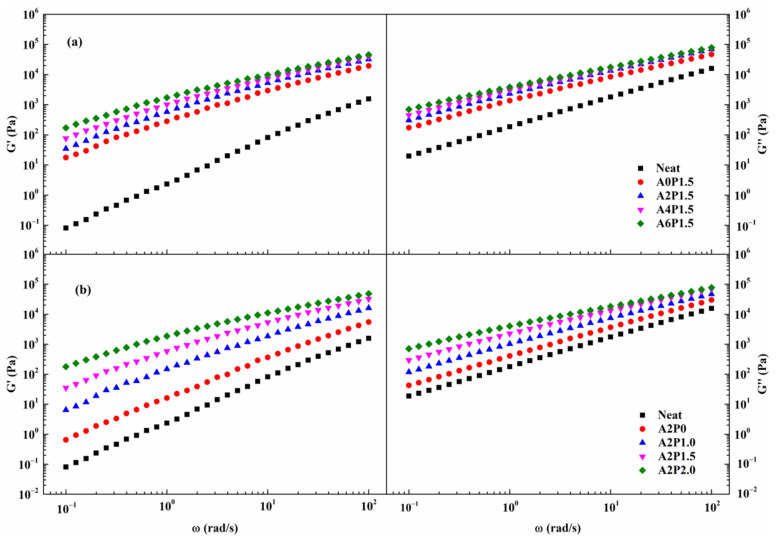
G′ and G″ variations with frequency for composite modified binders: (**a**) composite modified binders with various contents of APAO; (**b**) composite modified binders with various contents of PPA.

From Figure 6, it is obvious that the shear storage modulus G′ and shear loss modulus G″ increase as the frequency increases. This phenomenon signifies that asphalt binder exhibits more stiff and elastic characteristics with increases in vehicle travel speed. The increases in frequency accelerate the rate of increase of G′, indicating that G′ is highly sensitive to frequency. The compound modification of APAO and PPA markedly enhances G′ and G″ in comparison to that of virgin binder.

It can be noted that the values of G′ and G″ vary with the concentration of APAO and PPA. As can be seen, both G′ and G″ increase significantly as the contents of APAO or PPA increase. Remarkably, the increment in G′ is much greater than that of G″, showing that G′ has a strong dependence on the APAO or PPA concentration. As mentioned above, APAO or PPA are contributing factors to the enhancement of the elastic component, which further strengthens the elastic behavior. Moreover, the value of G′ increases significantly when the APAO concentration is 2 wt.% and the PPA concentration exceeds 1.0 wt.%. Therefore, the PPA proportion should be optimized to enhance the synergistic modification effect of APAO.

#### 3.3.2. Temperature Sweep Tests

**Figure 7:**
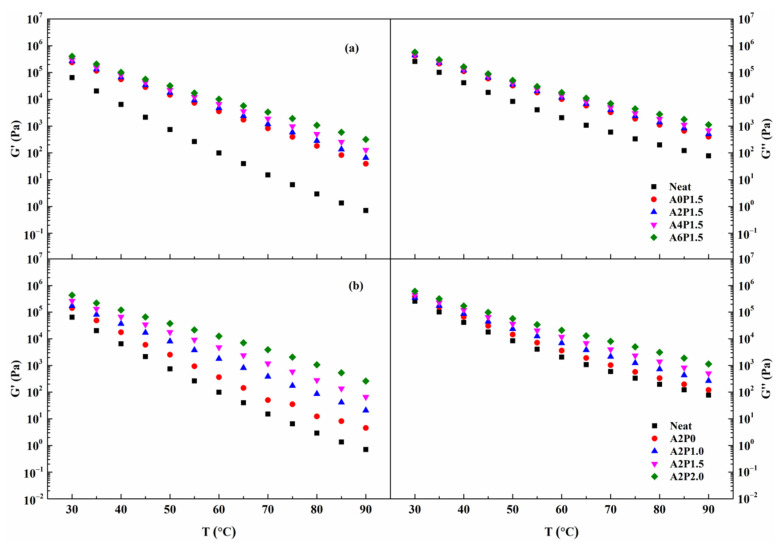
G′ and G″ variations with temperature for composite modified binders: (**a**) composite modified binders with various contents of APAO; (**b**) composite modified binders with various contents of PPA.

Temperature is the main factor that affects the rheological behavior of asphalt binders [46]. The temperature dependence of G′ and G″ of the compound modified binders is shown in Figure 7. Clearly, the parameters G′ and G″ show a decreasing trend with increasing temperature. Moreover, the reduced level of G′ is higher than that in G″ with increasing temperature, indicating that higher temperatures promote the transition of asphalt binder from an elastic to viscoelastic state. G″ always has a greater value than G′, revealing that the viscous component is more dominant. Thus, the elastic component in asphalt binder should be enriched to enhance its high-temperature resistance.

The increasing concentration of APAO and PPA contributes to a marked enhancement of G′ and G″. When the PPA content remains at 1.5 wt.%, increasing the concentration of APAO leads to an increment in G′ and G″, with the former experiencing a higher growth rate. This suggests that APAO strengthens the elastic behavior which represents a stronger high-temperature deformation resistance. Similarly, when the APAO proportion is 2 wt.%, an increase in PPA concentration also enhances the G′ and G″ values. The G′ value increases significantly when the content of PPA exceeds 1.0 wt.%, providing evidence that an appropriate amount of PPA benefits the reinforcement of anti-rutting performance of composite modified asphalt binder. Therefore, the enhancement of viscoelastic performances of asphalt binder is based on the interaction of APAO and PPA.

**Figure 8:**
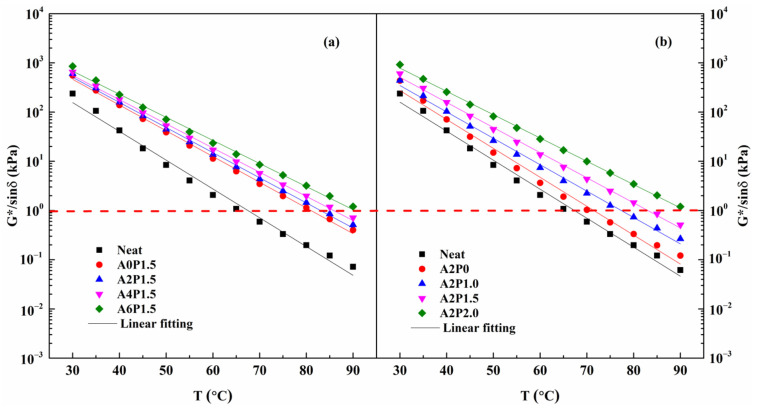
Variations in G*/sinδ with temperature for composite modified binders: (**a**) composite modified binders with various contents of APAO; (**b**) composite modified binders with various contents of PPA.

The rutting resistance index G*/sinδ is utilized to characterize the anti-rutting property of asphalt binders. Higher G*/sinδ signifies better resistance to permanent deformation. The calculated rutting index G*/sinδ values of all samples are displayed in Figure 8. As shown, G*/sinδ increases with increasing content of APAO, indicating that APAO benefits the anti-rutting property of the binder. Similarly, the rising concentration of PPA also leads to an increasing trend of G*/sinδ. It is worth noting that PPA has a greater contribution to the increment in G*/sinδ. Moreover, the failure temperatures of asphalt binders are acquired through calculation when the G*/sinδ value is 1.0 kPa. Failure temperatures of all tested samples are given in Table 7. Obviously, the failure temperatures increase with the addition of APAO and PPA. The incorporation of APAO markedly improves the failure temperature, with an increase of 9.38 °C for A6P1.5 compared to A0P1.5. However, PPA has a greater contribution to the increase in failure temperature. This result can be demonstrated by the fact that the failure temperature of A2P2.0 is 19.66 °C higher than that of A2P0. The failure temperature of modified binder A2P2.0 is 91.15 °C, which is 23.67 °C higher than that of virgin binder. Therefore, the combined effect of APAO and PPA can significantly improve the high-temperature properties of composite modified binder, with PPA contributing more to this improvement.

#### 3.3.3. Creep and Recovery Behavior

The rutting index G*/sinδ has a disadvantage in that it cannot reflect the delayed elastic deformation and recoverability of asphalt binder and thus fails to simulate real loading conditions [47]. The Federal Highway Administration (FHWA) proposed the MSCR test to more precisely evaluate the rheological behavior of modified binders at high temperatures [48]. A good correlation has been found between the non-recoverable creep compliance of the MSCR test and the anti-rutting property of asphalt mixtures [49,50,51]. Therefore, the MSCR test is employed to explore the influence of APAO and PPA on the high-temperature properties of the neat binder.

**Figure 9:**
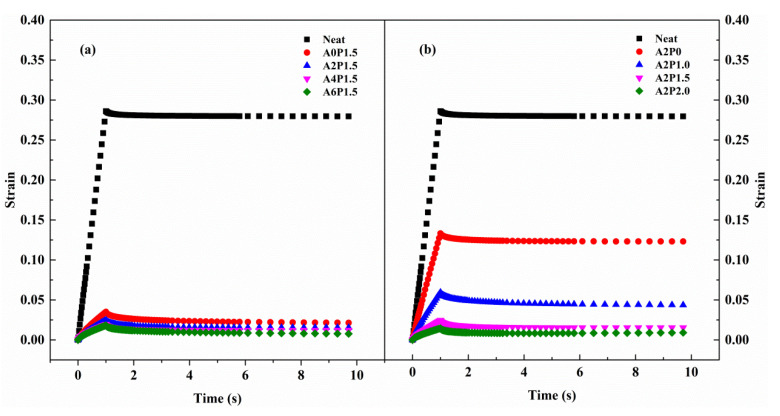
A creep–recovery cycle of the composite modified binder at 0.1 kPa and 60 °C: (**a**) composite modified binders with various contents of APAO; (**b**) composite modified binders with various contents of PPA.

The samples aged by RTFOT were subjected to the MSCR test at 60 °C and shear stress levels of 0.1 kPa and 3.2 kPa, respectively. For instance, Figure 9 presents the variation of strain with time for all samples at 0.1 kPa. As can be seen, one cycle includes a one-second creep stage and a nine-second recovery stage. The strain level increases with increases in loading time in the creep stage. In the stage of recovery, the strain recovers instantly when the loading stress is removed, and the recovery rate decreases with the loading time. A residual strain still exists after each cycle of creep and recovery. The above results reflect the viscoelastic–plastic performance of asphalt binder. The elastic strain recovers rapidly, while the viscous strain recovers slowly after the loading stress is eliminated. The residual strain represents the permanent deformation. It is notable that the neat binder has the maximum residual strain while the modified binder A2P2.0 has the minimum residual strain. This phenomenon demonstrates that the combined effect of APAO and PPA can lower the permanent deformation of neat binder.

**Figure 10:**
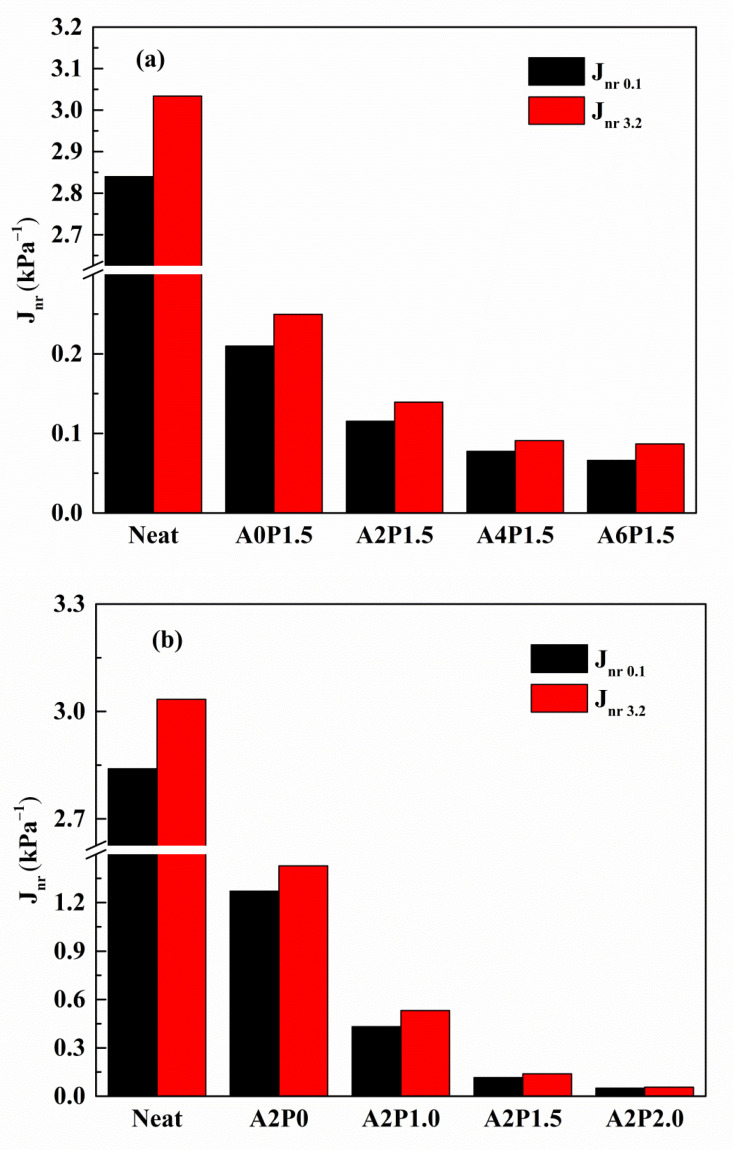
The J_nr_ of composite modified binders under 0.1 kPa and 3.2 kPa at 60 °C: (**a**) composite modified binders with various contents of APAO; (**b**) composite modified binders with various contents of PPA.

**Figure 11:**
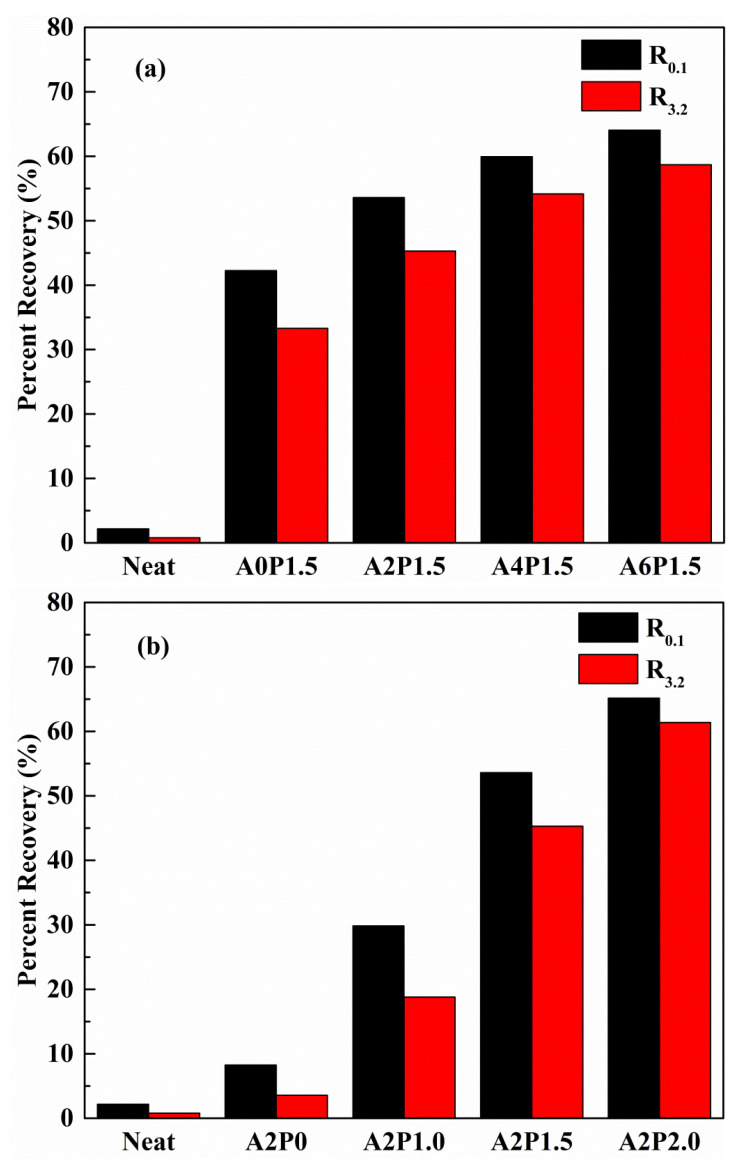
The R of composite modified binders under 0.1 kPa and 3.2 kPa at 60 °C: (**a**) composite modified binders with various contents of APAO; (**b**) composite modified binders with various contents of PPA.

Two main parameters of average non-recoverable creep compliance (J_nr_) and average percent recovery (R) were proposed by the FHWA to evaluate the high-temperature viscoelastic characteristics of the modified binder. The J_nr_ and R are defined as the permanent deformation and elasticity indexes of asphalt binder, respectively. The higher the R value is, the lower the J_nr_ value is, indicating that the anti-rutting property of asphalt binder is better. Figure 10 and Figure 11 show the J_nr_ and R values of composite modified asphalt binders at different stress levels. As observed from Figure 10a and Figure 11a, the addition of 1.5 wt.% PPA alone decreases the J_nr_ and increases the R in comparison to neat binder, indicating that PPA reduces the permanent deformation of neat binder. Similarly, 2 wt.% APAO alone is also capable of enhancing the anti-rutting performance of neat binder, as shown in Figure 10b and Figure 11b. When the concentration of PPA remains constant, increasing content of APAO leads to a marked increase in the R value and a significant reduction in the J_nr_ value. Asphalt binders modified with a certain amount of APAO and increasing content of PPA also exhibit a similar increasing trend of the R value and decreasing trend of the J_nr_ value. Moreover, the R value increases by 51.50% and the J_nr_ value decreases by 68.48% as the APAO increases from 0 wt.% to 6 wt.% under 0.1 kPa stress. The R value increases by 6.89 times and the J_nr_ value decreases by 96.06% as the PPA increases from 0 wt.% to 2 wt.%. Relative to APAO, PPA contributes more to the decrease in J_nr_ and the increase in R. Therefore, this suggests that PPA contributes more to the improvement of the elasticity of asphalt binder.

The variations in J_nr_ and R are also affected by the applied stress level. The J_nr_ exhibits an increasing trend and the R presents a decreasing trend when the stress increases from 0.1 kPa to 3.2 kPa. This suggests that a higher stress level reduces the anti-rutting performance of the asphalt binder. Thus, an asphalt pavement with heavier traffic is prone to experiencing rutting stress. Furthermore, the R and J_nr_ values vary with APAO and PPA concentration. Taking the R value as an example, the R values of A0P1.5, A2P1.5, A4P1.5 and A6P1.5 decrease by 21.26%, 15.49%, 9.63% and 8.36%, respectively, due to the increased stress level. Similarly, the R values of A2P0, A2P1.0, A2P1.5 and A2P2.0 drop by 56.81%, 36.98%, 15.49% and 5.81%, respectively. The results of the data analysis reveal that the combined effect of APAO and PPA is helpful to mitigate the adverse impact of the increased stress level.

#### 3.3.4. Low-Temperature Creep Behavior

**Figure 12:**
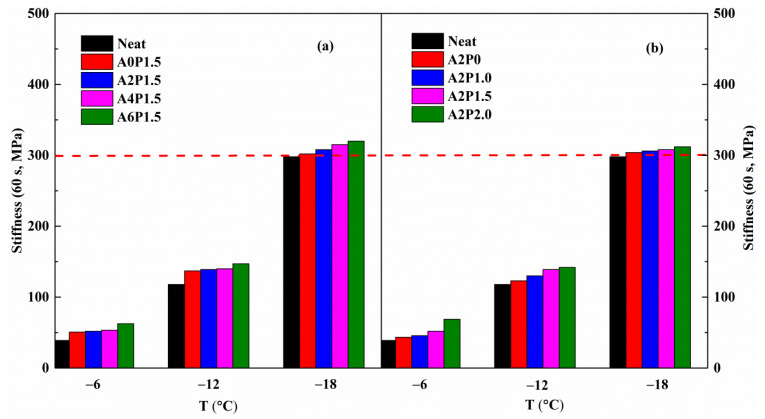
Creep stiffness variations with temperature for composite modified binders: (**a**) composite modified binders with various contents of APAO; (**b**) composite modified binders with various contents of PPA.

**Figure 13:**
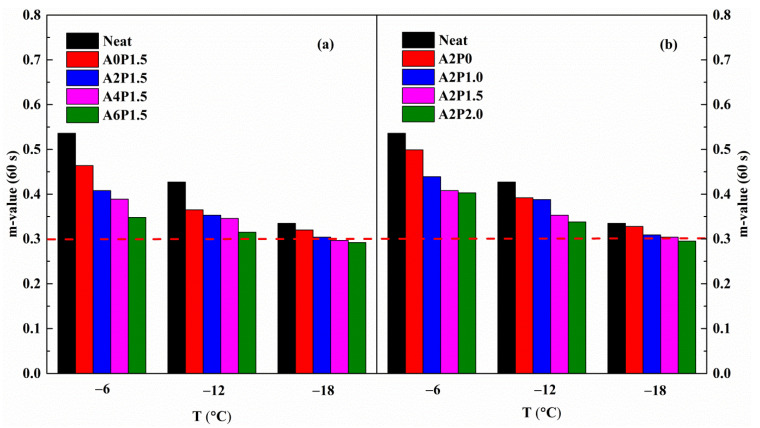
m-value variations with temperature for composite modified binders: (**a**) composite modified binders with various contents of APAO; (**b**) composite modified binders with various contents of PPA.

An asphalt pavement should be flexible enough to avoid cracking at low temperatures, which is dependent upon the characteristics of the asphalt binder. The Strategic Highway Research Program (SHRP) recommends the BBR test to assess the binder’s low-temperature properties. In the SHRP specification, the creep stiffness (S) and rate of creep (m-value) obtained under a 60 s loading are the two main parameters to identify the low-temperature properties of the binder. The creep stiffness represents the shrinkage stress of an asphalt pavement caused by temperature change, and the creep rate reflects the degree of stress reduction. Lower creep stiffness means less shrinkage stress due to a temperature drop, indicating better low-temperature crack resistance. Higher m-values represent a better stress dissipation property. It is well known that asphalt pavements with lower stiffness and higher m-value have better low-temperature cracking resistance performance. To investigate the influence of APAO and PPA on the low-temperature properties of neat binder, variations in stiffness and m-value are presented in Figure 12 and Figure 13.

As can be seen, the stiffness value increases and the m-value declines as the temperature drops from −6 °C to −18 °C. The increase in stiffness indicates that the binder becomes stiffer and has greater shrinkage stress, and the decrease in m-value suggests that the stress dissipation rate becomes smaller. The incorporation of APAO and PPA has a marked influence on the variations in stiffness and m-value. As observed, the stiffness of virgin binder presents an increasing trend and the m-value exhibits a decreasing trend as the concentration of APAO increases. This result indicates that APAO lowers the binder’s low-temperature performance. However, the increasing concentration of PPA further improves the stiffness and reduces the m-value, indicating that PPA decreases the low-temperature property on the basis of APAO modified asphalt binder. The stiffness increases by 23.47% as the APAO content rises from 0 wt.% to 6 wt.% at −6 °C, and the stiffness increases by 58.76% as the PPA content increases from 0 wt.% to 2.0 wt.%. The results show that the deterioration in the low-temperature performance caused by APAO is less than that by PPA.

The stiffness of asphalt binder is less than 300 MPa and the m-value is higher than 0.3, which meet the requirements of the SHRP specification [52]. The neat binder can meet the requirement at −12 °C, but fails at −18 °C. After adding APAO and PPA, all composite modified asphalt binders fulfill the specifications at −12 °C. As expected, the composite modified binders fail to meet the requirement at −18 °C. It is clear that the composite modified binder has comparable low-temperature performance to the virgin binder. The aforementioned results indicate that the low-temperature grade is not reduced by the compound modification of APAO and PPA. Thus, the low-temperature PG grade of APAO/PPA modified binders can reach −22 °C, which meets the requirements of most areas in China. Therefore, it is clear that the compound modification of APAO/PPA is detrimental to the stiffness and m-value of asphalt binder, although it does not reduce the low-temperature PG grade.

#### 3.3.5. Fatigue Behavior

**Figure 14:**
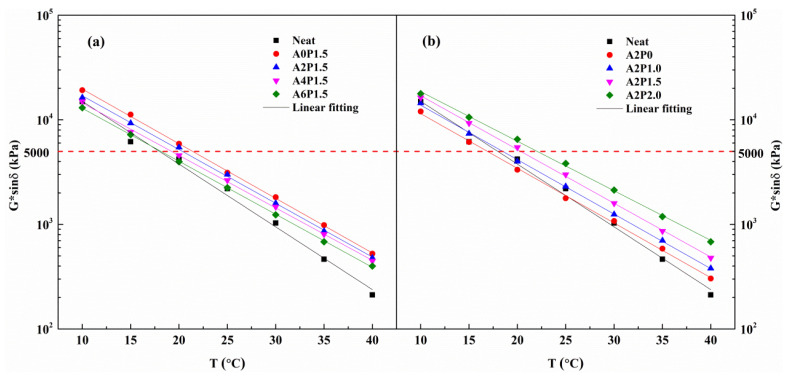
G *sinδ variations with temperature for composite modified binders: (**a**) composite modified binders with various contents of APAO; (**b**) composite modified binders with various contents of PPA.

Fatigue cracking of asphalt pavement has become an important stress factor influencing the long-term service performance of pavements. There are many factors contributing to fatigue cracking, such as climate change, binder properties, pavement design and structure and traffic volume. Among these influencing factors, it is well established that the anti-fatigue performance of asphalt binder plays a significant role in controlling the fatigue properties of asphalt mixtures [53]. The Superpave fatigue index G *sinδ, corresponding to the dissipated energy, is utilized to characterize the fatigue resistance of asphalt binder. Thus, the RTFOT-PAV aged specimens were subjected to a temperature sweep test to assess the binder’s fatigue resistance, the G *sinδ values of which are displayed in Figure 14.

As observed, G *sinδ values decrease with increases in temperature. In the intermediate temperature range, a small G *sinδ value indicates a better anti-fatigue performance of the binder. As observed from Figure 14a, it is obvious that the neat binder has a lower G *sinδ value, which means the neat binder has better fatigue resistance. After adding 1.5 wt.% PPA, the G *sinδ value becomes larger, suggesting that the PPA reduces the anti-fatigue property of asphalt binder. On the other hand, the G *sinδ value declines with increasing APAO content, indicating that APAO strengthens the fatigue resistance of the asphalt binder. However, there is an intersection between the fitting curves of neat binder and modified binder A6P1.5, which makes it difficult to determine the fatigue temperature. Moreover, Figure 14b shows that the modified asphalt binder with 2 wt.% APAO has a better anti-fatigue resistance performance than neat binder, and the G *sinδ value increases with increases in PPA content. To quantitatively investigate the effects of compound modification of APAO and PPA on fatigue properties, the fatigue temperatures are determined.

In accordance with the SHRP specification, the fatigue temperatures of asphalt binder are calculated when the G *sinδ value reaches the criterion of 5000 kPa [54]. The specific values of fatigue temperature are shown in Table 8. Obviously, A2P0 has the lowest fatigue temperature, followed by the neat binder. The increasing content of APAO leads to reductions in fatigue temperature, indicating that APAO can promote the anti-fatigue property of the binder. In contrast, increasing content of PPA increases the fatigue temperature, which indicates that PPA reduces the anti-fatigue property of the asphalt binder. The modified binder A6P1.5 has a lower fatigue temperature than A2P2.0. The combined effects of APAO and PPA on the fatigue resistance of the binder depend on their respective concentrations. Therefore, the APAO and PPA content should be optimized to guarantee the fatigue resistance of composite modified binder.

### 3.4. Storage Stability

Modified asphalt binder requires excellent storage stability during high-temperature storage, pumping, transportation and construction [55]. However, phase separation often occurs due to the incompatibility between polymers and asphalt binder. The principal reason for this is that the neat binder and polymer have great differences in density, molecular weight, polarity and solubility parameters. The phase separation leads to performance degradation in terms of anti-rutting, anti-cracking and fatigue resistance of modified binder, hindering the practical application of modified asphalt binder [56,57]. Therefore, the storage stability of composite modified binders containing various contents of APAO and PPA is evaluated by the storage test.

The temperature differences between the top and bottom parts in the storage test of modified binders are calculated, as shown in Table 9. To ensure the storage stability of the modified binder at a high temperature, a temperature difference of less than 2.5 °C is required. It is observed that the modified binder A0P1.5 is stable at elevated temperatures. After adding APAO, the temperature difference increases with increasing concentration of APAO, and phase separation becomes predominant for contents higher than 4 wt.%. Modified binder A2P0 has good storage stability due to the uniform dispersion of APAO in asphalt binder. After PPA is added, the softening point difference is less than 2.5 °C when PPA content reaches 2.0 wt.%. Such a phenomenon reveals that PPA has little influence on the storage stability of modified binders. In conclusion, the APAO proportion should be maintained at 2 wt.% to ensure storage stability.

It is generally believed that good compatibility between binder and polymer is essential to ensure the excellent stability of polymer modified binder. In view of the accuracy, the rheological method has a unique advantage in determining compatibility because it is sensitive to the differences between polymers and asphalt binder. At present, rheological methods such as master curves, black curves, Ham diagrams, and Cole–Cole diagrams are employed to characterize the compatibility of modified binders [54,55,58,59]. Among the above approaches, the Cole–Cole diagrams have proven to be the most effective method for determining compatibility. Thus, the compatibilities of APAO/PPA modified asphalt binder with various amounts of APAO and PPA were studied by a Cole–Cole diagram. The Cole–Cole plots comprise two variables, η′ and η″, which are separated from the complex viscosity η*. The compatibility is determined by the shape of the Cole–Cole diagram. A more symmetrical parabola represents better compatibility, while an asymmetrical parabola indicates poor compatibility.

**Figure 15:**
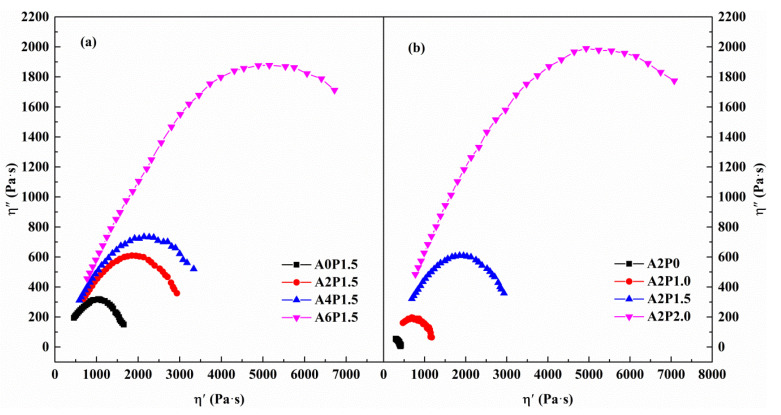
Cole–Cole diagrams of composite modified binders: (**a**) composite modified binders with various contents of APAO; (**b**) composite modified binders with various contents of PPA.

The Cole–Cole diagrams of APAO/PPA modified binders at 60 °C are displayed in Figure 15. It is clear that the η″ values of samples initially increase and then decrease with increases in η′, with a peak value appearing on the curve. The modified asphalt binders with various concentrations of APAO and PPA exhibit different curve shapes, which mainly depend on the effects of the modifier on the viscoelastic constituents of the asphalt binder. The left part and right part of the Cole–Cole diagram characterize the elastic and viscous performances of the binder, respectively. As shown in Figure 15a, the modified binder A0P1.5 exhibits a symmetrical shape, indicating better compatibility between PPA and neat binder. After APAO is added, the curves gradually transition from right to left with increasing content of APAO, showing that APAO can enhance the elastic components of modified asphalt binder. The curve is mostly concentrated on the left side when the APAO content reaches 6 wt.%, suggesting that the elastic component occupies a dominant position. Moreover, the increase in PPA content also leads to improvement in elastic constituents, as demonstrated by the transition of curves from right to left. Remarkably, modified binder A2P1.5 shows the most symmetrical shape, followed by the modified binder A4P1.5. When the APAO concentration reaches 6 wt.% or the PPA content reaches 2.0 wt.%, the Cole–Cole curves show a tendency to deviate from the semicircle. This demonstrates that 2 wt.% APAO and 1.5 wt.% PPA are the optimal concentrations for the preparation of modified asphalt binder with superior compatibility.

**Figure 16:**
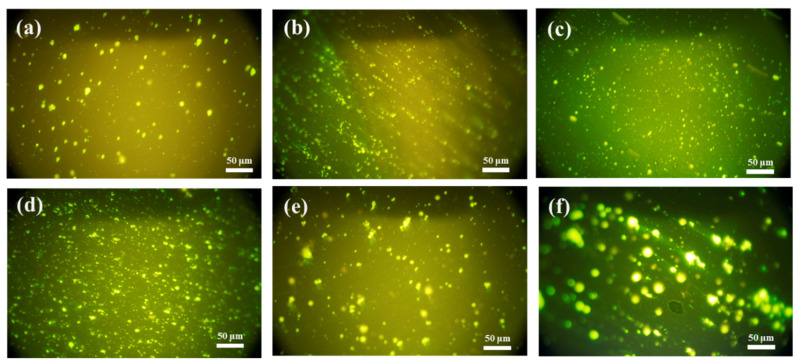
Morphology of tested samples (×400). (**a**) A2P0; (**b**) A2P1.0; (**c**) A2P1.5; (**d**) A2P2.0; (**e**) A4P1.5; and (**f**) A6P1.5.

It is important to investigate the distribution of polymer in asphalt binder to explore the behavior of polymer modified binders. The morphologies of APAO/PPA modified binders were observed by a fluorescence microscope. Figure 16 presents the fluorescence images of tested samples where the APAO particles are bright yellow and the asphalt binder phase is black. As observed in Figure 16a, the APAO particles appear in the form of spherical particles and are sparsely dispersed in the binder. However, the dispersion state of APAO changed significantly after the addition of PPA. The particle size decreases markedly and the distribution becomes much more uniform with increasing content of PPA, as shown in Figure 16b–d. This indicates that PPA is conducive to dispersing the APAO particles, and proves that PPA strengthens the compatibility between APAO and binder. Furthermore, Figure 16c,e,f show that the particle size of APAO increases and the distribution tends to aggregate as the APAO concentration increases from 2 wt.% to 6 wt.%. When the APAO proportion exceeds 4 wt.%, the boundary between the APAO and the asphalt binder phase is obvious and the dispersion worsens, revealing that potential phase separation may occur. This phenomenon corroborates the results of the Cole–Cole plots.

### 3.5. FTIR Spectra

**Figure 17:**
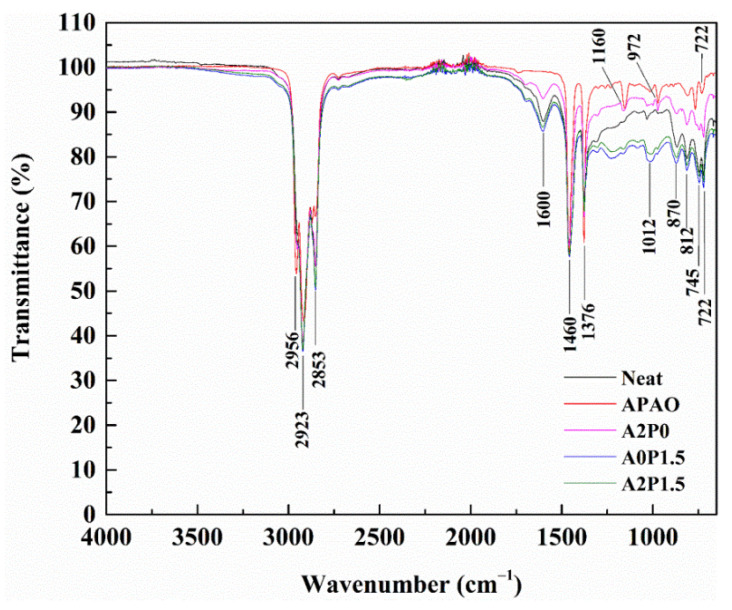
Infrared spectra of tested samples.

The analysis conducted above shows the variations of the conventional and rheological performances of modified binder after modification with APAO/PPA. More importantly, the microstructure of the modified binder primarily affects its macroscopic rheological behavior. FTIR has been widely used as an efficient method to evaluate the variations in chemical bonds and structures of modified binders. To ascertain the modification mechanism of the APAO/PPA modified binder, the infrared spectra of neat binder and APAO/PPA modified binder were acquired. Figure 17 displays the FTIR spectra of the modified binders.

It is observed that the typical characteristic peaks at 2956 cm^−1^, 2923 cm^−1^ and 2853 cm^−1^ denote the C-H stretching vibrations of aliphatic hydrogen. The absorption bands appearing at 1600 cm^−1^ correspond to the stretching vibrations of C=C. The C-H bending vibrations are located at 1460 cm^−1^ and 1376 cm^−1^. The characteristic absorption peaks within the 700–900 cm^−1^ range are the C-H bending vibrations of the aromatic ring.

The APAO is formed through the polymerization reaction of α-olefines, and contains a polymerized methylene group. The 1160 cm^−1^ and 972 cm^−1^ peaks represent the wagging and rocking vibration of C-H, respectively. The methylene group corresponds to the absorption band at 722 cm^−1^. The above peaks are considered to be typical characteristic peaks of APAO [7]. It is clear that there is no difference in absorption peaks between the neat binder and modified binder A2P0 in the infrared spectrum, indicating that APAO reacts with asphalt binder physically. However, after PPA is added, a new absorption band appears at 1012 cm^−1^, representing the P-O stretching vibration [17,60]. Masson et al. [61,62,63,64] showed that a phosphorylated product was generated by the reaction of asphalt binder with PPA. Ge et al. [65] pointed out that PPA caused the generation and disappearance of some functional groups. These results reveal that PPA reacts with asphalt binder chemically. In conclusion, APAO and PPA modification involves both physical and chemical interactions.

## 4. Conclusions

To simplify the production process, APAO and PPA were blended to produce a composite modified binder at a lower processing temperature without shearing and stabilizer. The influence of APAO/PPA on the conventional performance, temperature sensitivity, rheological property, storage stability, compatibility and microstructure of neat binder was studied. According to the above results, the primary findings and conclusions can be summarized as follows:

(1) The addition of APAO and PPA improves the high-temperature performance of asphalt binder, in which the values of softening point and viscosity are increased. The low-temperature properties and the temperature sensitivity of asphalt binder can be reduced through the compound modification of APAO and PPA.
(2) The frequency sweep test shows that the APAO/PPA strengthens the elastic behavior of composite modified asphalt binder under different traffic speeds. The temperature sweep test indicates that APAO/PPA improves the rutting resistance of asphalt binder by increasing the failure temperature. In the MSCR test, the increase in percent recovery and the decrease in non-recoverable creep compliance demonstrate that APAO/PPA improves the permanent deformation resistance of asphalt binder.
(3) Both APAO and PPA are detrimental to the stiffness and m-value of asphalt binder, while they do not reduce the low-temperature PG grade. The low-temperature PG grade of APAO/PPA modified binder can reach −22 °C, which meets the requirements of most areas in China. APAO can strengthen the fatigue resistance of the binder, while PPA reduces the anti-fatigue performance.
(4) A highly stable composite modified binder could be prepared by optimizing the content of APAO and PPA, as was confirmed by Cole–Cole plots and fluorescence images. The FTIR test reveals that both physical and chemical reactions occur during the modification process.
(5) Considering the composite modified binder’s rheological behavior and storage stability, the optimal dosage of APAO and PPA is 2 wt.% and 1.5 wt.%, respectively.

## Figures and Tables

**Table 1:** The recent studies on APAO and PPA modified asphalt binder.

Reference	Major Objective	Major Key Findings
Behnood, A. et al. [1]	Commonly used asphalt modifier	The commonly used polymers for asphalt binder modification can be classified into thermoplastic elastomers and plastomers.
Vargas, M.A. et al. [2]	Compatibility of PE and asphalt binder	A substantial phase separation occurs between the PE and the asphalt binder.
Fang, C., Luo, W.-Q., Zhu, C. et al. [3,4,5]	Preparation process	Polymers, such as SBS, PE, EVA, must be blended with asphalt binder by high-speed shearing equipment.
Zhu, J. et al. [6]	Polymer modification	Equipment investment and high-shearing temperatures increase the production cost of the modified asphalt binder.
Wei, J., Liu, N. et al. [7,8]	New polymer	APAO is a low molecular weight amorphous plastic material, which is highly compatible with binder.
Wei, J. et al. [7,9]	Compatibility of APAO and asphalt binder	APAO can be completely dissolved in asphalt binder when the APAO content is no more than 6 wt.%.
Kong et al. [10]	Processing temperature	Processing temperature of APAO modified binder can be controlled at 165 °C.
Yan et al. [11] and Liu et al. [12]	Effect of APAO on asphalt binder properties	APAO improved the high-temperature performances, storage stability and aging resistance of WTR modified binder.
Liu et al. [13]	Effect of APAO on asphalt binder properties	SBS/APAO modified binder had superior high- and intermediate-temperature properties and storage stability compared to SBS modified binder.
You et al. [14]	Effect of APAO on asphalt binder properties	APAO enhanced the high- and low-temperature property and storage stability of TB.
Yan et al. [15]	Effect of APAO on asphalt binder properties	APAO strengthened the elastic recovery and aging resistance and low-temperature cracking resistance of EVA modified binder.
Polacco, G. [16] and Zhang, F. [17]	Compatibility of PPA and asphalt binder	PPA has good compatibility with asphalt binder and has been widely applied in asphalt binder modification.
Venkat Ramayya, V. [18] and Jiang, X. [19]	Preparation process	PPA modified binder can be prepared by stirring at 150–160 °C instead of high-speed shearing.
Arnold, T.S. et al. [20]	Compatibility of PPA and asphalt binder	PPA does not separate from the asphalt phase when it modifies the asphalt binder without a stabilizer.
Alam, S. et al. [21]	Effect of PPA on asphalt binder properties	A small amount of PPA can markedly improve the Superpave performance grade of asphalt binder.
Behnood, A., Cao, W.D., Baldino, N. et al. [22,23,24,25]	Effect of PPA on asphalt binder properties	The useful temperature interval is extended by PPA.
Zhang, F., Gama, D.A., Xiao, F. et al. [26,27,28,29]	Effect of PPA on asphalt binder properties	PPA is also adopted to modify asphalt binder with another polymer to strengthen its rheological behavior and reduce the cost.
Zhang, F. et al. [27,30]	Effect of PPA on asphalt binder properties	PPA can enhance the compatibility between polymer and asphalt binder.

**Table 2:** Physical properties of neat binder.

Item	Neat Binder	Specifications
Softening Point (°C)	45.8	ASTM D36 [31]
Penetration (25 °C, 0.1 mm)	90	ASTM D5 [32]
Ductility (10 °C, cm)	>100	ASTM D113 [33]
Viscosity (135 °C, Pa·s)	0.348	ASTM D4402 [34]

**Table 3:** Physical properties of APAO.

Item	APAO-828	Specifications
Softening Point (°C)	161	ASTM D36
Penetration (25 °C, 0.1 mm)	22	ASTM D5
Viscosity (190 °C, Pa·s)	25	ASTM D4402
Density (23 °C, g/cm^3^)	0.87	ASTM D 1505 [35]
Glass Transition Temperature (°C)	−35	ASTM D3418 [36]
Molecular Weight (g/mol)	61,000	ASTM D 4001 [37]

**Table 4:** The composition and corresponding abbreviations of all modified binders.

Item	APAO (wt.%)	PPA (wt.%)	Item	APAO (wt.%)	PPA (wt.%)
Neat	0	0	A0P1.5	0	1.5
A2P0	2	0	A2P1.5	2	1.5
A4P0	4	0	A4P1.5	4	1.5
A6P0	6	0	A6P1.5	6	1.5
A0P1.0	0	1.0	A0P2.0	0	2.0
A2P1.0	2	1.0	A4P2.0	2	2.0
A4P1.0	4	1.0	A6P2.0	4	2.0
A6P1.0	6	1.0	A2P2.0	6	2.0

**Table 5:** The slope and intercept values of fitting curves.

Item	Slope	Intercept	R^2^
Neat	−3.4620	9.4445	0.9998
A0P1.5	−3.3734	9.2860	0.9999
A2P1.5	−3.2720	9.0385	0.9999
A4P1.5	−3.2116	8.8976	0.9998
A6P1.5	−3.1872	8.8566	0.9994
A2P0	−3.3957	9.2961	0.9994
A2P1.0	−3.3599	9.2391	0.9994
A2P1.5	−3.2720	9.0385	0.9999
A2P2.0	−3.2081	8.8975	0.9996

**Table 6:** The mixing and compaction temperatures for all samples.

Samples	Mixing Temperature (°C)	Compaction Temperature (°C)
Neat	150.90	139.69
A0P1.5	172.95	160.86
A2P1.5	179.64	166.99
A4P1.5	186.02	172.96
A6P1.5	194.04	180.64
A2P0	158.38	146.76
A2P1.0	169.56	157.51
A2P1.5	179.64	166.99
A2P2.0	189.07	175.90

**Table 7:** Failure temperatures of all tested specimens.

Samples	Failure Temperature (°C)
Neat	67.48
A0P1.5	81.00
A2P1.5	83.03
A4P1.5	85.84
A6P1.5	90.38
A2P0	71.49
A2P1.0	77.35
A2P1.5	83.03
A2P2.0	91.15

**Table 8:** Fatigue temperatures of all tested specimens.

Samples	Fatigue Temperature (°C)
Neat	17.96
A0P1.5	21.39
A2P1.5	20.33
A4P1.5	19.22
A6P1.5	18.12
A2P0	16.89
A2P1.0	18.47
A2P1.5	20.33
A2P2.0	22.04

**Table 9:** Softening point differences of composite modified binders.

Samples	Top Section (°C)	Bottom Section (°C)	Difference (°C)
A0P1.5	57.7	57.6	0.1
A2P1.5	61.8	61.5	0.3
A4P1.5	71.4	65.5	5.9
A6P1.5	74.1	67.6	6.5
A2P0	48.7	48.5	0.2
A2P1.0	54.7	54.4	0.3
A2P1.5	61.8	61.5	0.3
A2P2.0	67.8	66.5	1.3

## Data Availability

The data presented in this study are available on request from the corresponding author.
